# Evaluation of centralised and decentralised models of care during the 2020 Ebola Virus Disease outbreak in Equateur Province, Democratic Republic of the Congo: A brief report

**DOI:** 10.12688/f1000research.150755.2

**Published:** 2024-08-28

**Authors:** Emmanuel Lampaert, Justus Nsio Mbeta, Divya Nair, Maria Mashako, Anja De Weggheleire, Armand Sprecher, Rebecca M. Coulborn, Steve Ahuka-Mundeke

**Affiliations:** 1 Bureau Administratif et Liaison Intersection, Medecins Sans Frontieres, Kinshasa, Kinshasa, Democratic Republic of the Congo; 2 Ministère de la Santé, Kinshasa, Kinshasa, Democratic Republic of the Congo; 3 Centre for Operational Research, International Union Against TB and Lung Disease, 2 Rue Jean Lantier, Paris, 75001, France; 4 Médecins Sans Frontières MSF-OCB, Kinshasa, Kinshasa, Democratic Republic of the Congo; 5 Médecins Sans Frontières MSF-OCB, Brussels, 1050, Belgium; 6 Epicentre, Avenue Jean Jaurès, Paris, 75019, France; 7 Institut National de Recherche Biomédicale, Kinshasa, Kinshasa, Democratic Republic of the Congo

**Keywords:** Viral Haemorrhagic Fever, Central Africa, SORT IT, Outbreak, Epidemic response, Decentralized care, Operational Research, Ebola

## Abstract

**Background:**

Traditionally in the Democratic Republic of the Congo (DRC), centralised Ebola treatment centres (ETCs) have been set exclusively for Ebola virus disease (EVD) case management during outbreaks. During the 2020 EVD outbreak in DRC’s Equateur Province, existing health centres were equipped as decentralised treatment centres (DTC) to improve access for patients with suspected EVD. Between ETCs and DTCs, we compared the time from symptom onset to admission and diagnosis among patients with suspected EVD.

**Methods:**

This was a cohort study based on analysis of a line-list containing demographic and clinical information of patients with suspected EVD admitted to any EVD health facility during the outbreak.

**Results:**

Of 2359 patients with suspected EVD, 363 (15%) were first admitted to a DTC. Of 1996 EVD-suspected patients initially admitted to an ETC, 72 (4%) were confirmed as EVD-positive. Of 363 EVD-suspected patients initially admitted to a DTC, 6 (2%) were confirmed and managed as EVD-positive in the DTC. Among all EVD-suspected patients, the median (interquartile range) duration between symptom onset and admission was 2 (1-4) days in a DTC compared to 4 (2-7) days in an ETC (p<0.001). Similarly, time from symptom onset to admission was significantly shorter among EVD-suspected patients ultimately diagnosed as EVD-negative.

**Conclusions:**

Since <5% of the EVD-suspected patients admitted were eventually diagnosed with EVD, there is a need for better screening to optimise resource utilization and outbreak control. Only one in seven EVD-suspected patients were admitted to a DTC first, as the DTCs were piloted in a limited and phased manner. However, there is a case to be made for considering decentralized care especially in remote and hard-to-reach areas in places like the DRC to facilitate early access to care, contain viral shedding by patients with EVD and ensure no disrupted provision of non-EVD services.

## Introduction

Ebola virus disease (EVD) is a rare but deadly viral haemorrhagic fever with an average case fatality rate of 50% (ranging between 25-90%) among those infected. ^1^ Though vaccines and curative treatments are available, successful containment of an EVD outbreak is largely dependent on early detection, isolation, and treatment of cases. ^2^

The Democratic Republic of the Congo (DRC) in Central Africa has experienced 15 EVD outbreaks since 1976. ^3^ In the DRC, patients with EVD have been managed through an EVD-centric approach, at EVD treatment centres (ETCs) which are constructed in locations with large numbers of cases and which function parallel to the existing healthcare delivery system. However, recent outbreaks in the DRC and elsewhere revealed that local communities associated ETCs with remoteness and death and were reluctant to seek care from these facilities. ^4^^–^^7^ These beliefs led to delays in admission which in turn adversely impacted survival.

To address these concerns, a strategy of decentralization was piloted during the 11 ^th^ EVD outbreak, which occurred in 2020 in Equateur Province, DRC. Small teams, diagnostics, and supportive treatment services were deployed in existing local health centres in hard-to-reach areas to improve accessibility and allay communities’ apprehensions regarding ETCs. These were known as Decentralised Treatment Centres (DTCs). This approach aimed at reducing the risk of EVD transmission through early isolation of cases and improving patient outcomes through early access to diagnosis and supportive treatment while also ensuring that non-EVD health services continued to be provided to the communities.

The decentralized approach in the Equateur province has not yet been evaluated. The province merits attention in view of its large geographic expanse, tropical ecosystem conducive to re-emergence of EVD, presence of hard-to-reach pockets and a predominantly rural population which set it apart from most provinces of the DRC. Five of the fifteen outbreaks in the DRC have occurred in and around this province. ^3^

This study therefore aimed to report on the utilization of decentralized facilities and whether these facilities helped promote early admissions and early diagnosis during the 2020 EVD outbreak in Equateur. This information can be used in preparation for future outbreaks, in terms of resource allocation, training of human resources and provision of EVD management infrastructure at existing health centres in the country.

## Methods

### Study design

This was a retrospective cohort study making secondary use of data collected primarily for clinical purposes.

### Study setting

*General setting*

**Figure 1.**
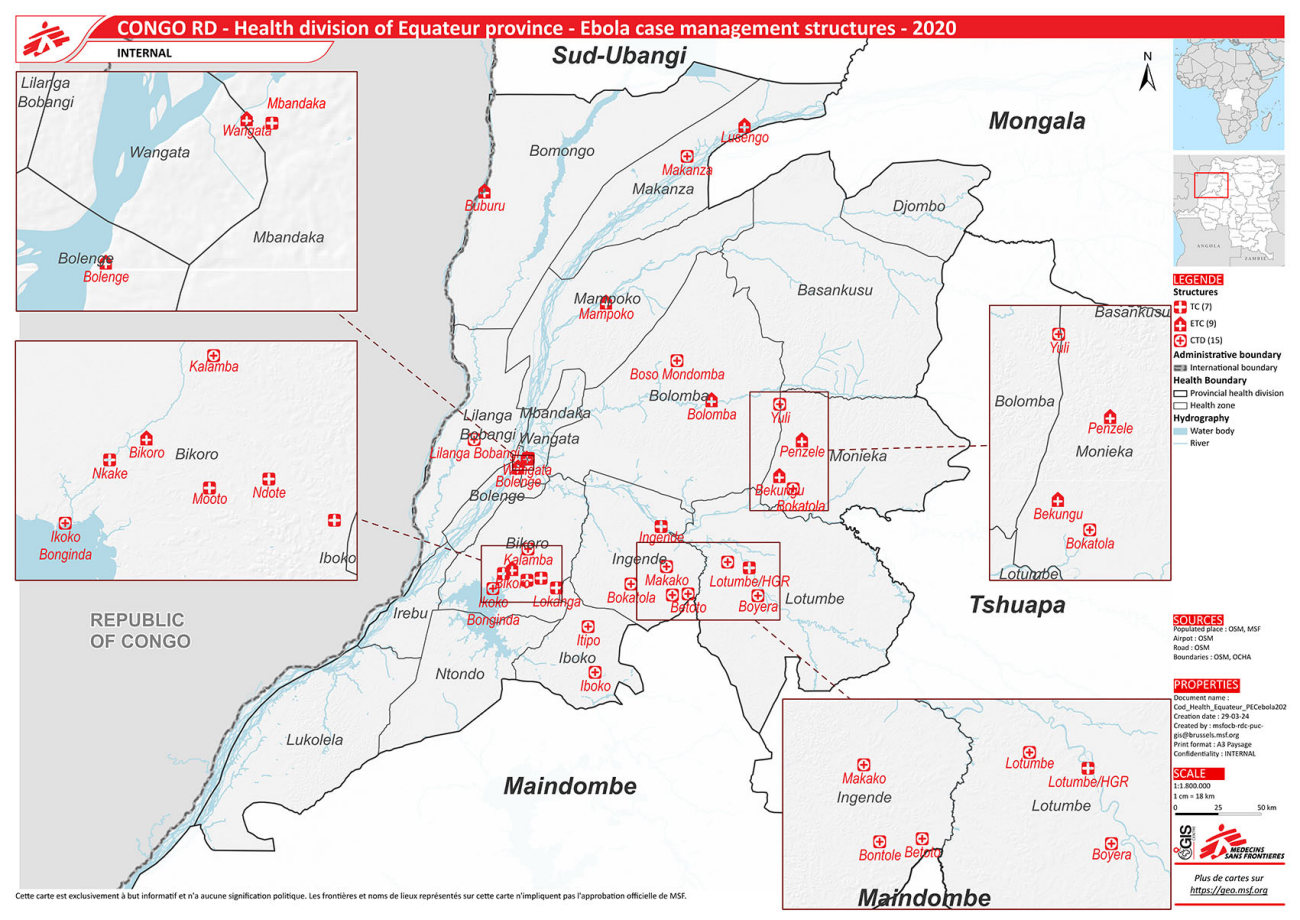
Health zones and locations of centralized and decentralized treatment centres during the 2020 Ebola Virus Disease Outbreak in Equateur Province, Democratic Republic of the Congo. (Source: Geographic Information System Centre, Médecins Sans Frontières). Abbreviations: ETC=Centralised Ebola treatment centre, CTD=Decentralised treatment centre, TC=Transit centre.

The DRC is the largest country in central Africa with a population of 112 million as of 2023. ^8^^,^^9^ It is one of the five poorest countries globally. ^8^ Equateur is one of its 26 administrative provinces with a population of 1.6 million and is divided into 18 health zones ( Figure 1). Each health zone provides services to a population of 100,000-200,000, and is further divided into health areas for every 10,000 population. ^10^

The DRC has a three-tier public health system: primary health centres in every health area, a secondary “General Reference Hospital” in each health zone and a tertiary “Provincial Hospital” in each provincial capital. Services in these facilities are provided on payment of user fees by the patients. ^10^ Mbandaka, the provincial capital of Equateur, is around 1200 km by road from the national capital of Kinshasa. Within the province, the Congo river system is the major channel for transport and there is limited road connectivity.

*Specific setting: EVD Outbreak of 2020*

The outbreak occurred in Equateur between 1 ^st^ June 2020 and 18th November 2020. ^11^ It produced 130 cases (119 confirmed and 11 probable), of which 75 recovered and 55 died. ^3^^,^^12^ Outbreak response was coordinated by the Ministry of Health (MOH) with technical support from multiple international aid organizations. Case definitions followed the World Health Organization recommendations. ^13^ All EVD care services were provided free of cost during the outbreak.

In the initial period, suspected cases were admitted to ETCs which were set up exclusively for EVD care. These centres were equipped with isolation units, diagnostic facilities, and advanced treatment modalities including monoclonal antibodies ( Table 1). A total of nine ETCs were newly constructed (mostly semi-temporary structures) during the outbreak ( Figure 1).

**Table 1.** Infrastructure and services offered at centralized and decentralized treatment centres in Equateur Province, Democratic Republic of Congo during the 2020 outbreak of Ebola Virus disease (EVD).

	Centralized Ebola treatment centres	Decentralized treatment centres
Main considerations for designation	• Near existing large (referral) hospitals where trained health care workers were available • Availability of physical infrastructure in terms of space for setting up an isolated advanced treatment facility	• Depending on epidemiological investigation, health centres located close to a cluster of cases were selected • Health centers located in densely populated areas were prioritized • Minimum infrastructure required in terms of a physical building with space for a triage and beds for isolating the suspected and probable EVD cases. • Concurrence of local community leaders was sought to ensure acceptance in the community
Location	Newly constructed during outbreak and located close to localities with large number of cases	Co-located with existing health centres in remote health areas
Mode of admission	1. Self-referral: Any EVD-suspected case who approached the centre for care 2. Referral of symptomatic EVD-contacts or EVD-contacts with a strong epidemiological link by health workers 3. Referral from decentralized treatment centres 4. Referral from transit centre	1. Self-referral: Any EVD-suspected case who approached the centre for care 2. Referral of symptomatic EVD-contacts or EVD-contacts with a strong epidemiological link by health workers
Human resources	• Medical doctors and nurses with expertise in EVD • Round the clock presence of experts- local and central expertise from MOH and/or external aid partners ^a^	• Nurses involved in primary health care service delivery (with basic orientation training in EVD) • Led by local MoH staff with technical support from experts if and when required
Bed capacity	Variable (based on patient load, epidemiological trends, and the transmission chain); 10 – 43 beds	Limited; average = 6 beds
EVD Diagnostic facilities	Yes (GeneXpert PCR in all ETCs with repeat of GeneXpert PCR at ETC Wangata for confirmation)	Sample collection locally with transport to ETC Wangata for GeneXpert PCR
Treatment facilities	Yes (Investigational treatment like monoclonal antibody therapeutics ^b^)	Only supportive treatment available and referral to ETC for advanced therapeutics (including monoclonal antibody treatment)
Isolation facilities	Yes	Yes
Biomedical waste management	Yes	Yes
Safe and dignified burial of dead bodies	Supported by the team of the ETC	Supported by the team of the DTC
Management of non-EVD cases	For EVD-negative cases, samples are sent to Kinshasa to rule out other febrile illness including malaria and other viral haemorrhagic fevers. EVD-negative cases are *transferred out to a different health care facility* for treatment	For EVD-negative cases, samples are sent to Kinshasa to rule out other febrile illness including malaria and other viral haemorrhagic fevers, and *treated within the same facility*

^a^ Includes Médecins Sans Frontières, The Alliance for Medical Action, and International Medical Corps

^b^ Inmazeb was approved by FDA during this outbreak (October 14 2020)

Seven transit centres (TCs) were also established in places where there was no testing capacity. Samples were taken from suspected cases and transported to ETCs. Patients were kept in isolation at the TCs while awaiting laboratory results. These TCs were also considered as centralized centres, functioning parallel to the existing medical system. Some of the TCs were converted into ETCs over time.

**Figure 2.**
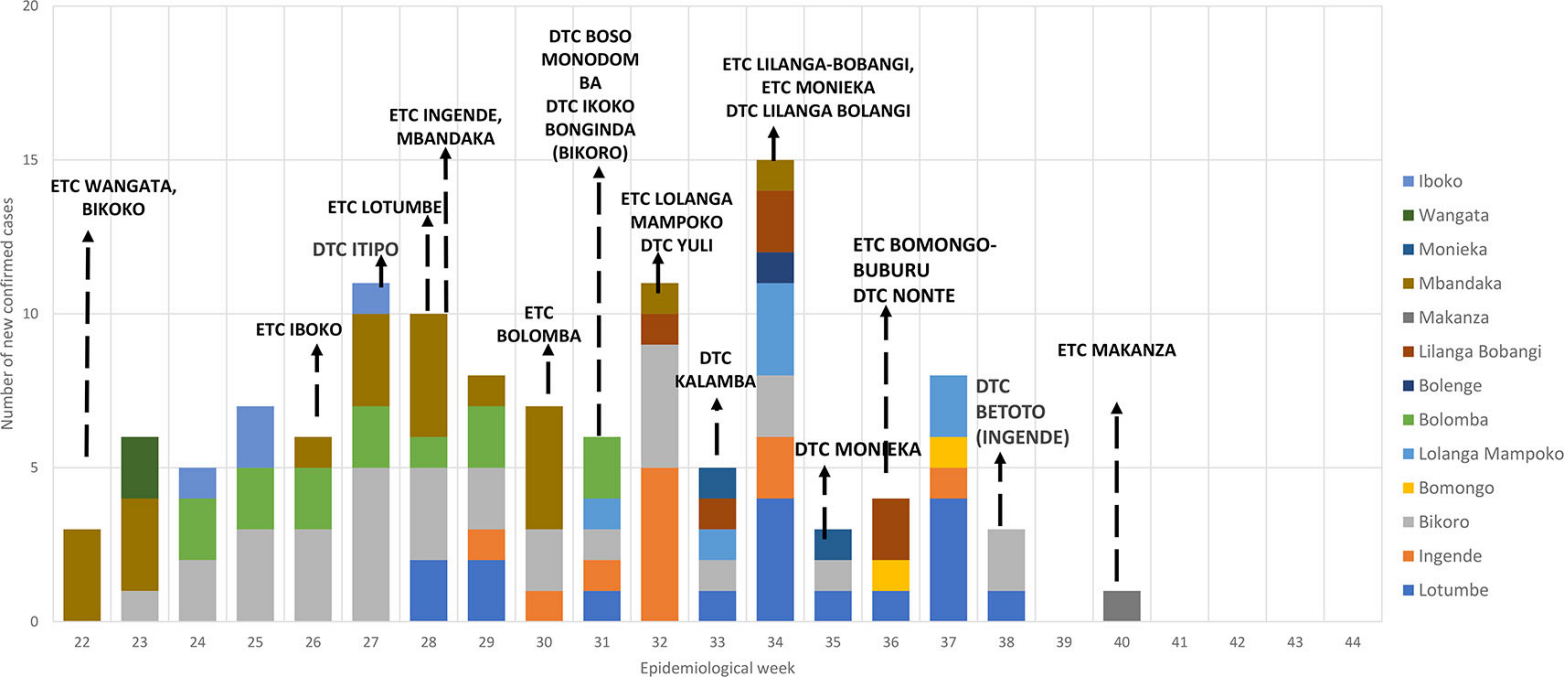
Graph showing the time trend of confirmed cases and the establishment of Centralised Ebola treatment centre (ETC) and Decentralised treatment centre (DTC) during the 2020 outbreak of Ebola Virus disease in Equateur Province, Democratic Republic of Congo.

As the outbreak progressed, suspected cases were reported from remote health zones and a decentralized approach was piloted. This approach was developed by the MOH in consultation with Médecins Sans Frontières (MSF). Based on contact tracing and epidemiological investigations, health areas where cases would be expected were identified. In these areas, the existing health centres were equipped to be decentralized treatment centres (DTCs). The first DTC started functioning in July 2020; 15 DTCs were established during the outbreak. The decision on designation of a facility as an ETC or DTC was made by the outbreak response team led by the MoH. Once a facility was selected to function as a DTC, the outbreak response team demarcated a triage area and an isolation area, ensured supply of basic personal protective equipment and apparatus required for sample collection-transport and for providing supportive treatment for suspected cases were available and established a biomedical waste management system, including identification of safe burial spaces in consultation with the local community. Table 1 provides a description of the services provided at the ETCs and DTCs. Figure 2 shows the time trend of confirmed cases and the establishment of ETC and DTCs, based on available data.

### Study population

All patients with suspected EVD admitted to any EVD health facility during the 2020 EVD outbreak in Equateur were included.

### Data collection, sources, and analysis

During the outbreak, the Médecins Sans Frontières (MSF) team under directions of the MOH developed a common form to collect the basic details of patients admitted as suspected cases to any EVD health facility. A healthcare worker was identified as a focal person in each facility and was responsible for data collection and updation of the form on a daily basis. A supervisor was identified within the MSF team who was responsible for collating the paper forms from a group of facilities and digitising them into a linelist. At the time of digitization, the supervisor would check for data completeness and whenever possible coordinate with the facility focal person to retrieve missing data from the facility treatment records and update the linelist. The compiled line-list of patients with suspected EVD admitted to any EVD health facility constituted the data source.

Data on patient demographic characteristics, clinical characteristics at the time of presentation, final diagnosis, and treatment outcomes were extracted from the line-list and analysed using STATA (version 16.0, StataCorpLLC, College Station, Texas, USA). R is an open-access software which can be used to conduct the same analysis.

The outcomes of interest were time to admission, time to diagnosis and final treatment outcomes. Time to admission was calculated as the duration between the date of symptom onset and date of first admission. Time to diagnosis was calculated as the duration between the date of symptom onset and date of the first positive PCR test (for confirmed cases) or date of the earliest negative PCR test (for non-EVD cases). Date of treatment initiation was not recorded in the line-list, and therefore time taken to initiate treatment could not be assessed. For patients transferred from one facility to another, the outcome reported at the final EVD health facility was considered as the final outcome.

Time to admission and diagnosis were summarized as medians with inter-quartile ranges and compared between the two types of facilities (i.e., ETC versus DTC) using Mann Whitney-U test. Final outcomes between the two were compared using the Chi-square test. A p-value < 0.05 was considered statistically significant.

Handling of missing data: The number of observations with missing data are reported for key variables. Since the outcome variables (time to diagnosis and time to admission) were non-parametric, imputation techniques and sensitivity analysis to assess impact of missingness in these variables were not performed. Statistical tests were performed after omitting the observations with missing data for the outcome variables pertaining to the test.

## Results

**Figure 3.**
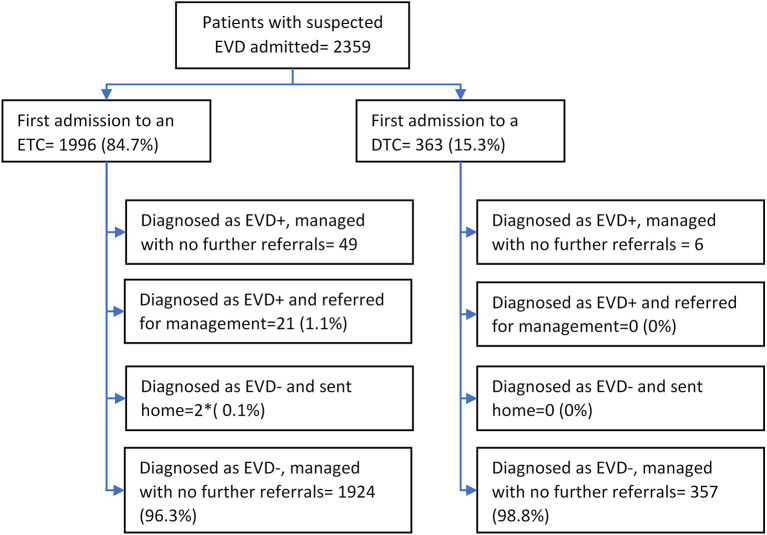
Flow of patients with suspected Ebola virus Disease (EVD) at centralized and decentralized EVD treatment centres in Equateur Province, Democratic Republic of the Congo, during the 2020 EVD outbreak. *These two patients were diagnosed as EVD-negative in the first ETC but were eventually confirmed as EVD-positive in the next ETC that they consulted. ETC=Centralised Ebola treatment centre, DTC=Decentralised treatment centre, EVD = Ebola Virus Disease, EVD+=EVD-positive; EVD-=EVD-negative.

There were 2359 line-listed unique patients suspected of having EVD. Of these, the type of EVD health facility visited first was an ETC for 1996 (85%) patients and a DTC for 363 (15%) patients. Of the 1996 patients with suspected EVD who were initially admitted to an ETC, 72 (4%) were confirmed as EVD-positive. Of the 363 patients with suspected EVD who were initially admitted to a DTC, 6 (2%) were confirmed as EVD-positive in the same health facility and remained there for care ( Figure 3).

Table 2 shows the demographic and clinical characteristics of patients with suspected EVD based on the type of EVD health facility first visited. The age and gender distribution were not significantly different between the two types of facilities. In total, 895 (45%) patients with suspected EVD who first visited an ETC and 137 (38%) who first visited a DTC were not vaccinated. At the time of admission, certain signs or symptoms were reported by a significantly higher proportion of patients with suspected EVD who visited an ETC first compared to a DTC, for example: fatigue (75% vs 54%), muscle pain (35% vs 20%), breathlessness (13% vs 4%), bleeding (7% vs 3%), and dysphagia (7% vs 3%). Apart from EVD, the most common final diagnosis included malaria in 954 (48%) patients first admitted to an ETC and 57 (16%) patients first admitted to a DTC.

**Table 2.** Demographic and clinical characteristics of patients with suspected Ebola Virus Disease (EVD) admitted to centralised Ebola treatment centres and decentralised treatment centres during the 2020 EVD outbreak in Equateur Province, Democratic Republic of the Congo.

Characteristics	Type of facility visited first				P-value
	Centralised Ebola treatment centre		Decentralised treatment centre		
	n	(%)	n	(%)	
**Total**	1996	(100)	363	(100)	
**Age (in years)**					0.518
0-4	348	(17.4)	69	(19.0)	
5-14	449	(22.5)	76	(20.9)	
15-29	418	(20.9)	78	(21.5)	
30-44	370	(18.5)	77	(21.2)	
45-59	242	(12.1)	35	(9.6)	
60 and above	169	(8.5)	ret	(7.2)	
Not recorded	0	(0.0)	2	(0.6)	
**Gender**					0.152
Male	1010	(50.6)	165	(45.5)	
Female	986	(49.4)	190	(52.3)	
Not recorded	0	(0.0)	8	(2.2)	
**Vaccination against EVD**					<0.001
Vaccinated prior to admission	71	(3.6)	4	(1.1)	
Vaccinated after admission	4	(0.2)	0	(0.0)	
Unvaccinated	895	(44.8)	137	(37.7)	
Vaccinated but timing unknown	515	(25.8)	24	(6.6)	
Vaccination status unknown	511	(25.6)	198	(54.5)	
**Signs and symptoms at admission** ^a^					
Fever	1639	(84.3)	290	(80.1)	0.050
Fatigue	1459	(74.9)	196	(54.2)	<0.001
Vomiting	1006	(51.9)	176	(48.6)	0.250
Diarrhoea	713	(36.7)	131	(36.2)	0.849
Muscle pain	684	(35.2)	74	(20.4)	<0.001
Breathlessness	252	(12.9)	16	(4.4)	<0.001
Bleeding	143	(7.4)	10	(2.8)	0.001
Dysphagia	140	(7.2)	11	(3.0)	0.003
Hiccups	49	(2.5)	8	(2.2)	0.724
Conjunctivitis	27	(1.4)	4	(1.1)	0.665
**Final diagnosis**					NA ^b^
EVD-positive	70	(3.5)	6	(1.7)	
EVD-positive and Malaria	2	(0.1)	0	(0.0)	
Malaria	954	(47.8)	57	(15.7)	
Typhoid fever	5	(0.3)	1	(0.3)	
Respiratory Tract Infection	18	(0.9)	5	(1.4)	
Intestinal parasitosis	69	(3.5)	2	(0.6)	
Others ^c^	296	(14.8)	20	(5.5)	
Not recorded	582	(29.2)	272	(74.9)	
**Final EVD status**					0.056
Confirmed EVD	72	(3.6)	6	(1.6)	
Probable EVD ^d^	2	(0.1)	2	(0.6)	
Suspected EVD ^d^	76	(3.8)	12	(3.3)	
Non EVD	1843	(92.3)	343	(94.5)	
Not recorded	3	(0.2)	0	(0.0)	

EVD = Ebola Virus Disease

^a^ A patient could have multiple symptoms

^b^ Not applicable: Chi-square or Fisher’s exact test could not be applied due to the small numbers in cells

^c^ Other diagnosis included anaemia, fever of unknown origin, malnutrition and those entries in which “Other pathologies” was mentioned without specifying a diagnosis

^d^ Final status remained as “Probable EVD” or “Suspected EVD” in these patients: Reason is not clear from the available data and may be a data entry error or due to the fact that the patient left or died before a final diagnosis could be established.

Table 3 shows a comparison of pre-diagnostic delays based on the type of facility visited first. When all patients with suspected EVD were considered, the duration between symptom onset and admission to an EVD health facility was significantly shorter among those first admitted in a DTC (Median: 2 days, Interquartile range [IQR]: 1-4 days) compared to an ETC (Median: 4 days, IQR: 2-7 days). Similarly, the duration between symptom onset and diagnosis was significantly shorter among those first admitted in a DTC (Median: 3 days, IQR: 2-6 days) compared to an ETC (Median: 4 days, IQR: 2-7 days).

**Table 3.** Comparison of pre-diagnostic delays in suspected and confirmed Ebola Virus Disease (EVD) patients admitted to centralised Ebola treatment centres and decentralised treatment centres during the 2020 EVD outbreak in Equateur Province, Democratic Republic of the Congo.

EVD patients and time periods	Type of facility visited first						P-value
	Centralised Ebola treatment centre			Decentralised treatment centre			
	N ^a^	Duration (in days)		N	Duration (in days)		
		Median	(IQR) ^b^		Median	(IQR) ^b^	
**All suspected cases of EVD**							
Symptom onset to first admission	1978	4	(2-7)	352	2	(1-4)	<0.001
Symptom onset to diagnosis	1753	4	(2-7)	229	3	(2-6)	<0.001
**Confirmed cases of EVD**							
Symptom onset to first admission	72	5	(2-8)	6	3	(2-14)	0.727
Symptom onset to diagnosis	72	6	(3.5-8)	6	3	(2-14)	0.342
**Non-EVD cases**							
Symptom onset to first admission	1903	4	(2-7)	346	2	(1-4)	<0.001
Symptom onset to diagnosis	1681	4	(2-7)	223	3	(2-6)	<0.001

^a^ N represents the number of entries with valid dates for calculating duration.

^b^ IQR = inter quartile range.

Among patients with suspected EVD who were later confirmed to be EVD-positive, there was no significant difference in the time to admission and time to diagnosis based on the type of facility ( Table 3).

Among the patients with suspected EVD for which the final status was EVD-negative, the duration between symptom onset and admission among those first admitted to a DTC (Median: 2 days, IQR: 1-4 days) was significantly shorter than among those who were first admitted to an ETC (Median: 4 days, IQR: 2-7 days). Also, the time to diagnosis was significantly shorter among EVD-negative patients first admitted to a DTC compared to an ETC ( Table 3).

The final outcomes of the 78 EVD-positive patients are shown in Table 4. Among the 72 EVD-positive patients first admitted in an ETC, 60 (83%) were cured and 10 (14%) died. All six EVD-positive patients first admitted in a DTC were cured.

**Table 4.** Comparison of final outcomes of patients with confirmed Ebola Virus Disease (EVD) based on type of care facility initially visited during the 2020 EVD outbreak in Equateur Province, Democratic Republic of the Congo.

Final outcome	Total (N=78)		Type of facility visited first				P-value
			Centralised Ebola treatment centre (N=72)		Decentralised treatment centre (N=6)		
	n	(%)	n	(%)	n	(%)	
							0.757
Died	10	(12.8)	10	(13.9)	0	(0.0)	
Cured	66	(84.6)	60	(83.3)	6	(100.0)	
Lost to follow-up	1	(1.3)	1	(1.4)	0	(0.0)	
Transferred to other ETC	1	(1.3)	1	(1.4)	0	(0.0)	

## Discussion

To our knowledge, this is the first study exploring the decentralised model of care piloted during the 2020 EVD outbreak in the Equateur province of DRC. The study has three key findings. First, one out of seven patients with suspected EVD was first admitted to a DTC. Second, DTCs managed to reduce the time to admission diagnosis in all patients with suspected EVD (including some diagnosed later as EVD-negative). Third, 3% of all patients with suspected EVD were confirmed to have EVD. There were 12 EVD deaths in ETCs and none in a DTC.

The piloting of the DTC model marks a paradigm shift in outbreak control in the region – from a mostly EVD-centric approach to a more community-centric approach. ^14^^–^^17^ The DRC’s “Strategic response plan for the EVD outbreak: 2018” calls for strengthening existing heath facilities and empowering the existing health workforce to conduct efficient EVD triage, maintain continuity of EVD and non-EVD care, and take healthcare closer to communities so that individuals can seek care early. ^16^

The study has certain limitations. Since the dates of treatment initiation were missing for the majority of the patients, we could not evaluate the time taken to initiate treatment at ETCs compared to DTCs. Confounders like severity of illness (cycle threshold values) and the geographic proximity of patients to an EVD health facility might have impacted the time to admission, but we were unable to adjust for these due to the non-availability of data. These parameters should be meticulously documented in future outbreaks to enable a comprehensive evaluation of the DTC model. Given the small number of cases in this outbreak, we are unable to comment on whether the reduction in time to admission led to a difference in outcomes among patients first admitted to a DTC compared to an ETC. We were also unable to conduct qualitative interviews among patients and caregivers in these facilities, which could have provided in-depth insights into patient and provider perspectives around care seeking and delivery during the outbreak.

Despite these limitations, this study has important implications, more so because this is the first study exploring a decentralised model in the Equateur Province. Only 15% of the patients with suspected EVD in the 2020 outbreak first sought care in a DTC. This could be due to the fact that the DTCs were piloted one month into the outbreak in a limited and phased manner. Therefore, the ETCs bore the brunt of cases during the outbreak.

The DTCs appear able to reduce the time taken to admit and diagnose (EVD or non-EVD) patients with suspected EVD. This has two implications. First, DTCs could provide diagnosis and care to patients with other conditions during the outbreak. In countries like the DRC, a wide spectrum of febrile illnesses like malaria and viral haemorrhagic fevers are prevalent. ^18^^–^^20^ As care provision for these illnesses has been disrupted during previous EVD outbreaks in this region, ^21^^,^^22^ the establishment of DTCs might help overcome this issue. Second, the median time to admission was three days in the DTCs which was slightly lower than that reported in previous outbreaks in the DRC. ^23^^,^^24^ A reduction in time to admission and diagnosis among confirmed EVD patients could be crucial for initiating early EVD specific treatment and thus reducing mortality. ^1^^,^^25^

All six of the patients with confirmed EVD who first visited a DTC were diagnosed and cured at the same facility (i.e., the one initially visited). These patients might have had milder forms of disease which did not require referral. While this represents too small a number from which to draw firm conclusions on the effect of decentralised care on patient outcomes among those EVD-positive and could also be influenced by the severity of illness in those presenting to the DTC, it is an encouraging finding.

There is need to look at this model critically. Only 3% of all patients with suspected EVD admitted to a treatment centre were eventually diagnosed as EVD-positive. The 2020 outbreak resulted in 130 cases, among which 55 died. ^3^ However, only 78 patients (10 of whom died) were admitted to an EVD health facility. The rest of the patients were identified during contact tracing but could not be located or brought to a facility. The majority of deaths happened in the community, which indicates that severely ill patients who needed urgent care either did not seek care or could not be provided with care. More needs to be done to ensure that people have access to timely diagnostics and medical care. A qualitative exploration of the circumstances which led to these community deaths might be useful to understand why these individuals did not or could not access facility-based care. Also, since <5% of the patients admitted with suspected EVD were diagnosed as EVD, there is need for better screening to optimise resource utilization and infection control.

In conclusion, this assessment of the decentralised model of EVD care provision in Equateur was unable to draw inferences on the impact of the model on treatment outcomes due to the relatively small size of the 2020 outbreak and lack of data on potential confounding factors which could impact outcomes. Notwithstanding the limitations of this study, decentralized models of care offer an opportunity to potentially reduce community transmission of EVD and improve access to care for all diseases, especially in remote and hard-to-reach areas. At the same time, it is imperative to ensure availability of relevant, timely and quality assured data during any future outbreaks for monitoring the response and comprehensively assessing the utility of decentralized models in the context of the DRC.

### Ethical considerations

Ethical approval with waiver of informed consent was obtained from (a) National Ethics Committee of the School of Public Health, University of Kinshasa, DRC (Approbation Number: ESP/CE/115/2023 dated 04 August 2023), (b) Médecins Sans Frontières Operational Center Brussels Ethics Review Board (24 July 2023) and (c) Union Ethics Advisory Group, International Union against Tuberculosis and Lung Disease, Paris, France (EAG Number: 17/2023 dated 08 September 2023). Permission to access the line-list data was obtained from the MOH of the DRC.

### Reporting guidelines

This reporting of this study followed the *STROBE guidelines* .

Repository: STROBE checklist for ‘Evaluation of centralised and decentralised models of care during the 2020 Ebola Virus Disease outbreak in Equateur Province, Democratic Republic of the Congo: A brief report’. 10.6084/m9.figshare.25983145.

Data are available under the terms of the Creative Commons Attribution 4.0 International license (CC-BY 4.0).

## Open access statement

In accordance with WHO’s open-access publication policy for all work funded by WHO or authored/co-authored by WHO staff members, WHO retains the copyright of this publication through a Creative Commons Attribution IGO license ( http://creativecommons.org/licenses/by/3.0/igo/legalcode) which permits unrestricted use, distribution and reproduction in any medium provided the original work is properly cited.

## Disclaimer

There should be no suggestion that WHO endorses any specific organization, products or services. The views expressed in this article are those of the authors and do not necessarily reflect those of their affiliated institutions. The use of the WHO logo is not permitted.

## Data Availability

Figshare: Evaluation of centralized and decentralized models of care during the 2020 Ebola Virus Disease outbreak in Equateur Province, Democratic Republic of the Congo: A brief report. https://doi.org/10.6084/m9.figshare.25634556. ^26^ The project contains the following underlying data: “Finaldataset F1000.xlsx” (Anonymised line-list of patients with suspected Ebola Virus Disease during the 2020 2020 Ebola Virus Disease outbreak in Equateur Province, Democratic Republic of the Congo). Data are available under the terms of the Creative Commons Attribution 4.0 International license (CC-BY 4.0).
