# Supplementary material for: Evaluation of centralised and decentralised models of care during the 2020 Ebola Virus Disease outbreak in Equateur Province, Democratic Republic of the Congo: A brief report
Source: F1000Res. 2024 Aug 28;13:642. Originally published 2024 Jun 17. [Version 2] doi: 10.12688/f1000research.150755.2 (PMC11406140; doi:10.12688/f1000research.150755.2)
Supplement: Peer review history

# Reviewer report: Reviewer response for version 2

**DOI:** 10.5256/f1000research.170852.r317862

**Published:** 2024-09-16

**Authors:** Francesco Branda

**Affiliations:** 1 Unit of Medical Statistics and Molecular Epidemiology, Università Campus Bio-Medico di Roma, Rome, Italy

Thanks to the authors for improving the work by following the suggestions made.

Is the work clearly and accurately presented and does it cite the current literature?

Partly

If applicable, is the statistical analysis and its interpretation appropriate?

Yes

Are all the source data underlying the results available to ensure full reproducibility?

Yes

Is the study design appropriate and is the work technically sound?

Yes

Are the conclusions drawn adequately supported by the results?

Partly

Are sufficient details of methods and analysis provided to allow replication by others?

Partly

Reviewer Expertise:

My research interests are diverse, spanning various domains such as data analytics, epidemic intelligence systems, and public health risk studies. To address these questions, I developed novel methods that combine techniques from mathematical modelling, and statistical inference (including AI and Machine Learning). My work focuses to epidemiological and statistical consulting in hospital settings, applying statistical and molecular methods in clinical settings, and analyzing climate-sensitive diseases like Dengue and Chikungunya and outbreaks and pandemics such as SARS-CoV-2, Mpox, and Ebola.

I confirm that I have read this submission and believe that I have an appropriate level of expertise to confirm that it is of an acceptable scientific standard.

# Reviewer report: Reviewer response for version 2

**DOI:** 10.5256/f1000research.170852.r317861

**Published:** 2024-09-03

**Authors:** Nlandu Roger Ngatu

**Affiliations:** 1 Department of Public Health, Kagawa University, Miki, Japan

I have no comment.

Is the work clearly and accurately presented and does it cite the current literature?

Yes

If applicable, is the statistical analysis and its interpretation appropriate?

Yes

Are all the source data underlying the results available to ensure full reproducibility?

Yes

Is the study design appropriate and is the work technically sound?

Yes

Are the conclusions drawn adequately supported by the results?

Yes

Are sufficient details of methods and analysis provided to allow replication by others?

Yes

Reviewer Expertise:

Environmental and occupational health; Global health; Infectious diseases.

I confirm that I have read this submission and believe that I have an appropriate level of expertise to confirm that it is of an acceptable scientific standard.

# Reviewer report: Reviewer response for version 1

**DOI:** 10.5256/f1000research.165351.r304876

**Published:** 2024-08-06

**Authors:** Nlandu Roger Ngatu

**Affiliations:** 1 Department of Public Health, Kagawa University, Miki, Japan

The authors compared time from the onset of EVD symptoms to admission and diagnosis in suspected EVD patients between ETCs and DTCs in a retrospective cohort study conducted in D.R. Congo. They found that the duration between onset of EVD symptoms and admission was significantly lower in suspected patients admitted at DTCs compared to those admitted in ETCs. 

 The manuscript is well written, and data are well presented. I have a few comments in relation to the methodology. 

 1. Study design: authors analyzed existing data from the outbreak that occurred in Equateur, D.R. Congo, in 2020. They should mention it as being a Retrospective cohort study.

 2. Data collection, sources and analysis: 

  - Authors should specify the outcome variable(s) 

     used in this study.

  - Authors stated that they used Stata software to analyze the data. They also mention R software. So, I wonder why "R" after using Stata? What was the purpose of using R software?

 I would be glad if authors can provide answers to the two comments.

Is the work clearly and accurately presented and does it cite the current literature?

Yes

If applicable, is the statistical analysis and its interpretation appropriate?

Yes

Are all the source data underlying the results available to ensure full reproducibility?

Yes

Is the study design appropriate and is the work technically sound?

Yes

Are the conclusions drawn adequately supported by the results?

Yes

Are sufficient details of methods and analysis provided to allow replication by others?

Yes

Reviewer Expertise:

Environmental and occupational health; Global health; Infectious diseases.

I confirm that I have read this submission and believe that I have an appropriate level of expertise to confirm that it is of an acceptable scientific standard.

# Response

**Published:** 2024-08-22

**Authors:** Divya Nair

**Affiliations:** Center for OperationalResearch, International Union against Tuberculosis and Lung Disease UK, Paris, Paris, France

We thank the esteemed reviewer for the comments. The following are responses to the two comments, as requested:

 1. **Study design**: authors analysed existing data from the outbreak that occurred in Equateur, D.R. Congo, in 2020. They should mention it as being a Retrospective cohort study.

***Response:*** *We agree and have changed the design to “Retrospective cohort study” in “Study design” section of the revised manuscript.*

 2. Data collection, sources and analysis:  

 - Authors should specify the outcome variable(s) used in this study.

 - Authors stated that they used Stata software to analyse the data. They also mention R software. So, I wonder why "R" after using Stata? What was the purpose of using R software?

***Response**:* *Thank you for the suggestion. We have listed the outcome variables in “Data collection, sources, and analysis” section of the revised manuscript.*

*Regarding R software, the F1000 Open Data, Software and Code Guidelines website (https://f1000research.com/for-authors/data-guidelines), mention that “Where third-party proprietary software has been used, a non-proprietary, Open Source alternative software should be suggested by the author to allow for the replication of the analysis or research by all readers”. Therefore, we have mentioned that “R is an open-access software which can be used to perform the same analysis” as an alternative to the proprietary software (Stata) that we used. Since this was a journal requirement, we would like to retain the statement in the manuscript.*

# Reviewer report: Reviewer response for version 1

**DOI:** 10.5256/f1000research.165351.r304877

**Published:** 2024-08-02

**Authors:** Francesco Branda

**Affiliations:** 1 Unit of Medical Statistics and Molecular Epidemiology, Università Campus Bio-Medico di Roma, Rome, Italy

**Summary:**

 This brief report evaluates centralized and decentralized models of care during the 2020 Ebola virus disease (EVD) outbreak in Equateur Province, Democratic Republic of Congo. The study compared the time from symptom onset to admission and diagnosis of patients with suspected EVD at centralized Ebola treatment centers (ETCs) versus decentralized treatment centers (DTCs) experienced during the outbreak. The main results were:

 1. 15% of patients with suspected EVD were admitted to a DTC for the first time.

 2. The time to admission and diagnosis was significantly shorter for patients admitted to DTCs for the first time compared to CTEs.

 3. Only 3% of all patients with suspected EVD were confirmed to have EVD.

 4. There were no deaths from EVD in CDIs, compared with 12 deaths in CTEs.

 The authors conclude that decentralized care models can help reduce community transmission and improve access to care, especially in remote areas, although further research is needed given the small size of the outbreak.

**Evaluation:**

 1. Is the study design appropriate and is the work technically sound? Yes, the cohort study design using secondary analysis of clinical data is appropriate for the research questions. The statistical methods used are sound.

 2. Are the methods and analyses detailed enough to allow replication by others?

 In part. More detail could be provided on:

 - Specific criteria used to designate a facility as an ETC or DTC.

 - Process for creating DTCs (e.g., how existing facilities were selected/equipped).

 - Data cleaning and quality control procedures for the list of data lines.

 - Handling of missing data

 3. If data is available, is the source and origin of the data clearly indicated?

 Yes, the source of the data (list of MOH lines) is clearly indicated and a link to the anonymized dataset is provided.

 4. Are the conclusions drawn adequately supported by the results?

 In part. The conclusions are generally supported, but some caveats could be stated more explicitly:

 - The small number of confirmed EVD cases limits conclusions about outcomes.

 - Potential confounding factors not taken into account (e.g., disease severity, geographic proximity).

 - Lack of qualitative data on patients' and providers' perspectives.

 5. Is the article comprehensibly presented and written in standard English?

 Yes, the article is well written and clearly presented.

**Recommendations for improvement:**

 1. Provide more methodological details as indicated above to improve reproducibility.

 2. Expand discussion of limitations, particularly regarding the small number of confirmed cases and potential confounding factors.

 3. Include, if possible, a brief qualitative component (e.g., interviews with health care providers) to provide context on the implementation of the DTC model.

 4. Clarify whether sensitivity analyses were conducted to assess the impact of missing data.

 5. Consider adding a figure showing the time trend of DTC establishment and number of cases to illustrate the phase-in.

 6. Provide more detail on deaths in the community that occurred outside of facilities, if available, to put the results in context.

 7. Discuss more explicitly in the conclusions the implications and recommendations for future outbreaks.

Is the work clearly and accurately presented and does it cite the current literature?

Partly

If applicable, is the statistical analysis and its interpretation appropriate?

Yes

Are all the source data underlying the results available to ensure full reproducibility?

Yes

Is the study design appropriate and is the work technically sound?

Yes

Are the conclusions drawn adequately supported by the results?

Partly

Are sufficient details of methods and analysis provided to allow replication by others?

Partly

Reviewer Expertise:

My research interests are diverse, spanning various domains such as data analytics, epidemic intelligence systems, and public health risk studies. To address these questions, I developed novel methods that combine techniques from mathematical modelling, and statistical inference (including AI and Machine Learning). My work focuses to epidemiological and statistical consulting in hospital settings, applying statistical and molecular methods in clinical settings, and analyzing climate-sensitive diseases like Dengue and Chikungunya and outbreaks and pandemics such as SARS-CoV-2, Mpox, and Ebola.

I confirm that I have read this submission and believe that I have an appropriate level of expertise to confirm that it is of an acceptable scientific standard, however I have significant reservations, as outlined above.

# Response

**Published:** 2024-08-22

**Authors:** Divya Nair

**Affiliations:** Center for OperationalResearch, International Union against Tuberculosis and Lung Disease UK, Paris, Paris, France

*We thank the esteemed reviewer for the recommendations for improvement. We would like to mention that this article is submitted under the ‘Brief Report’ category of the journal, and has a **word limit restriction of 2500 words**. Therefore, we have tried to address the concerns to the best of our ability within the constraints of the word limits.*

1. Provide more methodological details as indicated above to improve reproducibility. More detail could be provided on:
  - Specific criteria used to designate a facility as an ETC or DTC. ***Response: **As mentioned in the manuscript, the approach of decentralisation was piloted for the first time in the Equateur province. The decision on designation of a facility as an ETC or DTC was made empirically by the outbreak response team led by the MoH in consultation with the various aid partners. While there was no standard guideline document for these decisions, the following were the considerations. We have now added a row in Table 1 explaining these.*
  - Process for creating DTCs (e.g., how existing facilities were selected/equipped). ***Response: **The main considerations for selecting DTCs is outlined in the revised Table 1. Once a facility was selected to function as a DTC, the outbreak response team d* *emarcated a triage area and an isolation area, e* *nsured supply of basic personal protective equipment and apparatus required for sample collection-transport and for providing supportive treatment for suspected cases were available and e* *stablished a biomedical waste management system, including identification of safe burial spaces in consultation with the local community.* *These have been enumerated in the revised version of the manuscript under the “Specific setting” section.*
  - Data cleaning and quality control procedures for the list of data lines. ***Response**: During the outbreak, the MSF team (under directions of the MOH) developed a common form to collect the basic details of patients admitted as suspected cases to any EVD health facility. A healthcare worker was identified as a focal person in each facility and was responsible for data collection and updation of the form on a daily basis. This person was responsible for ensuring the quality and completeness of the data. MSF supervisors were responsible for electronic entry from paper forms on to an MS Excel file, known as the ‘Common linelist’. At the time of digitization, the supervisor would check for data completeness and whenever possible coordinate with the facility focal person to retrieve missing data from the facility treatment records and update the linelist. During the outbreak, this linelist was used primarily to know the occupancy rate of facilities and the vaccination status of suspected cases and these variables were the focus of quality control processes during the outbreak.* *We have included a brief description of this process in the revised version of the manuscript in the ‘Data collection, sources, and analysis’ section. We have also highlighted the quality issues and the need for good quality data in Discussion section.  *
  - Handling of missing data.**** ***Response: **The number of observations with missing data are reported for key variables. Since the outcome variables (time to diagnosis and time to admission) were non-parametric, imputation techniques and sensitivity analysis to assess impact of missingness in these variables were not performed. Statistical tests were performed after omitting the observations with missing data for the outcome variables pertaining to the test. This has been added under the “Data collection, sources, and analysis” section of the revised manuscript* 
2. Expand discussion of limitations, particularly regarding the small number of confirmed cases and potential confounding factors. The conclusions are generally supported, but some caveats could be stated more explicitly: The small number of confirmed EVD cases limits conclusions about outcomes. Potential confounding factors not taken into account (e.g., disease severity, geographic proximity). Lack of qualitative data on patients' and providers' perspectives. ***Response:** We agree with this suggestion and have tried to bring in the limitations in the “Conclusion” paragraph.*
3. Include, if possible, a brief qualitative component (e.g., interviews with health care providers) to provide context on the implementation of the DTC model. ***Response**: We agree that a qualitative component would have added value to this assessment. However, we are unable to conduct a qualitative inquiry at present because:* (1) *Since the outbreak in 2020, there have been massive staff turnovers and the relevant health care workers are no longer available in health zones which can be accessed by the principal investigator.* (2) *There is an ongoing political unrest and a Monkey Pox outbreak in the DRC which has led to diversion of human resources to conflict zones and MPox response. The principal investigator himself and the co-authors from DRC are involved in the response and shall not be able to conduct the qualitative interviews (seek ethical approvals, trace and interview the healthcare workers, analyse data).* *We have therefore cited this as a limitation. Also, we have referenced and cites a situational analysis of the first 90 days of the outbreak conducted by UNICEF (Reference 6), which provides some qualitative insights into the communities’ non-acceptance for ETCs and makes recommendations for decentralised care provision.*
4. Clarify whether sensitivity analyses were conducted to assess the impact of missing data. ***Response: **In this study, the key outcomes of interest which has missing data were time to diagnosis and time to admission. Both these variables were found to be non-parametric. To do a sensitivity analysis, we would need to use an imputation technique. We could apply a non-parametric imputation technique (hot-deck/ predictive mean matching) to generate their imputed values but we are unaware of any robust statistical package which would allow us to perform a Mann-Whitney test on the multiply imputed data sets. Therefore, we have refrained from using any imputation techniques in this study*
5. Consider adding a figure showing the time trend of DTC establishment and number of cases to illustrate the phase-in. ***Response:** We have now provided a figure titled “Graph showing the time trend of confirmed cases and the establishment of Centralised Ebola treatment centre (ETC) and Decentralised treatment centre (DTC) in Equateur Province, Democratic Republic of Congo during the 2020 outbreak of Ebola Virus disease” as Figure 2.*
6. Provide more detail on deaths in the community that occurred outside of facilities, if available, to put the results in context.**** ***Response:** We are unable to provide any more detail on deaths that occurred outside of facilities, apart from what is already stated in the manuscript. These deaths were retrospectively classified as Ebola deaths because of identified epidemiological links with confirmed cases of EVD. The reason for delayed notification of deaths and subsequent investigations could possibly be due to the occurrence of EVD cases in remote health zones in Northern parts of the province, which had no experience with EVD outbreaks in the past. However, this is speculative and we are unable to substantiate this with available data.*
7. Discuss more explicitly in the conclusions the implications and recommendations for future outbreaks. ***Response:** We have tried to expand the conclusion with inclusion of limitations and recommendations.*
